# Applications of Phyto-Nanotechnology for the Treatment of Neurodegenerative Disorders

**DOI:** 10.3390/ma15030804

**Published:** 2022-01-21

**Authors:** Tanima Bhattacharya, Giselle Amanda Borges e Soares, Hitesh Chopra, Md. Mominur Rahman, Ziaul Hasan, Shasank S. Swain, Simona Cavalu

**Affiliations:** 1 Innovation, Incubation & Industry (i-cube) Laboratory, Techno India NJR Institute of Technology, Udaipur 313003, India; 2 Department of Science & Engineering, Novel Global Community Educational Foundation, Hebersham, NSW 2770, Australia; 3 Department of Medicinal and Biological Chemistry, University of Toledo, 3000 Arlington Ave., Toledo, OH 43614, USA; giselleamanda.borgesesoares@rockets.utoledo.edu; 4 Department Pharmacology, Chitkara College of Pharmacy, Chitkara University, Punjab 140401, India; chopraontheride@gmail.com; 5 Department of Pharmacy, Faculty of Allied Health Sciences, Daffodil International University, Dhaka 1207, Bangladesh; mominur.ph@gmail.com; 6 Department of Biosciences & Centre for Interdisciplinary Research in Basic Sciences, Jamia Millia Islamia, Jamia Nagar, New Delhi 110025, India; zhasan.biochem@gmail.com; 7 Division of Microbiology and NCDs, ICMR-Regional Medical Research Center, Chandrasekharpur, Bhubaneshwar 751023, India; swain.shasanksekhar86@gmail.com; 8 Faculty of Medicine and Pharmacy, University of Oradea, 410087 Oradea, Romania

**Keywords:** nanomedicine, nanoparticles, phytomedicine, bioinformatics, neurodegenerative diseases

## Abstract

The strategies involved in the development of therapeutics for neurodegenerative disorders are very complex and challenging due to the existence of the blood-brain barrier (BBB), a closely spaced network of blood vessels and endothelial cells that functions to prevent the entry of unwanted substances in the brain. The emergence and advancement of nanotechnology shows favourable prospects to overcome this phenomenon. Engineered nanoparticles conjugated with drug moieties and imaging agents that have dimensions between 1 and 100 nm could potentially be used to ensure enhanced efficacy, cellular uptake, specific transport, and delivery of specific molecules to the brain, owing to their modified physico-chemical features. The conjugates of nanoparticles and medicinal plants, or their components known as nano phytomedicine, have been gaining significance lately in the development of novel neuro-therapeutics owing to their natural abundance, promising targeted delivery to the brain, and lesser potential to show adverse effects. In the present review, the promising application, and recent trends of combined nanotechnology and phytomedicine for the treatment of neurological disorders (ND) as compared to conventional therapies, have been addressed. Nanotechnology-based efforts performed in bioinformatics for early diagnosis as well as futuristic precision medicine in ND have also been discussed in the context of computational approach.

## 1. Introduction

Nanotechnological advancements in neurological science are projected to have a significant effect on the development of novel therapeutical strategies. The capability to produce nanoparticles in the same size domain as proteins has led to a wide range of applications in the biomedical field, as they can invigorate, respond to, and affect target cells and tissues to ensure the desired physiological reactions while diminishing undesirable results. In addition, nanotechnology can permit a more exact and ideal control of complex natural frameworks than customary pharmacological methodologies can, for example, the blood-mind obstruction (BBB). “Neurodegenerative diseases” (NDs) refers to a gathering of discontinuous or potentially familial conditions portrayed by the deficiency of neuronal subtypes over a long period. Alzheimer’s disease (AD) and Parkinson’s disease (PD) are amongst the most decimating illnesses of the twenty-first century [1,2,3,4,5,6].

Around 24 million individuals overall experience the ill-effects of dementia, with Alzheimer’s sickness representing 60% of cases [7]. Learning and memory issues occur in Alzheimer’s patients. Net cerebral decay, which demonstrates the deficiency of neurons and the presence of various neuronal extracellular plaques and intracellular neurofibrillary tangles, has been found in post-mortem examinations of human brain tissue, fundamentally in the front-facing and transient flaps, including the hippocampus [8]. Notwithstanding, although the pathophysiology of AD is known, the reason for which it occurs, and its trigger, is unknown. As a result, AD treatment is only based on symptomatic treatment of the disease. The degeneration of nigrostriatal dopaminergic neurons in the midbrain causes extreme side effects including hypokinesia, bradykinesia, unbending nature, and a resting quake [9].

Although Parkinson’s disease (PD) is the second most prevalent chronic and progressive non-degenerative illness (ND), it has an enormous social and economic effect on the great majority of people affected by it [10]. The ventral tegmental region and the substantia nigra of the brain produce less dopamine (DA) when dopamine neurons degenerate over time. Patients with Parkinson’s disease (PD) may have a wide range of symptoms including stiffness, tremor, and slowness of movement. In certain cases, it may be difficult to tell the difference between Parkinson’s disease and other conditions that have many of the same symptoms. As a result, the word “Parkinsonism” has come to describe a wide range of neurological conditions. Symptoms of Parkinsonism fall into two categories: primary and secondary. Idiopathic Parkinsonism, the most common kind of primary Parkinsonism, has no recognised aetiology. A variety of conditions may lead to secondary Parkinsonism, including progressive supranuclear palsy, multiple system atrophy, and others, the most frequent of which is drug-induced Parkinsonism following cerebrovascular illness. Vascular Parkinsonism is a distinct clinical entity that falls under the umbrella of Parkinsonism, although it has a variable natural history and a wide range of clinical manifestations. In addition, there is strong evidence for inclusion of clock genes in PD [10].

Dopamine agonists, such as levodopa, are currently utilized as first-line treatment for PD. However, as the disease advances, patients become less receptive to levodopa and disease progression continues. Profound mind incitement and foetal dopamine neuron transplantation have been examined as options in contrast to pharmacological consideration [11,12]. These medicines are currently suggestive, and do not appear to have the option to stop or counteract the progressing consumption of dopaminergic neurons [13,14,15]. Medication conveyance to the cerebrum remains a significant test in the treatment of AD and PD. The rise of new, useful treatment modalities for the treatment of NDs is an intriguing issue of examination at the present time [16]. Considering the various defensive hindrances that encompass the CNS, the development of effective therapeutics to treat patients with NDs is of crucial significance.

There are microvascular endothelial cells that line the cerebral capillaries of most mammals and other species with a well-developed CNS, which the BBB is composed of. Based on an average microvessel surface area of 150–200 cm^2^ per g of tissue, it is anticipated that a normal adult has a total surface area of 12–18 m^2^ [4]. The BBB serves as a barrier between the bloodstream and the central nervous system, protecting the brain’s parenchyma from exposure to potentially harmful substances carried by the bloodstream. Specialized ion channels and transporters combine in the BBB to keep the ionic composition optimal for neuronal and synaptic signalling processes. Potassium concentrations in the CSF and ISF, for example, are 2.5–2.9 mM. The plasma potassium concentration is 4.5 mM, regardless of whether the fluctuations are induced by illness or imposed artificially. Calcium, magnesium, and pH are all controlled by the BBB [17]. Neuronal excitability and macrophage movement across the BBB are regulated by calcium and potassium homeostasis [18].

The BBB’s limitation and the medication discharge energy that trigger fringe results are two significant realities about neurotherapeutics. Despite the prevalent attitude, it is presently realized that NDs can be multisystemic in nature, which represents different difficulties for future consideration. A course of a few harmful atomic and cell occasions, instead of a solitary pathogenic factor, causes the demise of specific kinds of neurons in NDs. Accordingly, nanotechnology can offer an expected answer for a significant number of these difficulties in the treatment of AD and PD by considering specific medication conveyance and improving the bioavailability as well as the viability of different medications and other bioactive specialists utilized in NDs [19].

## 2. Materials and Methods

Relevant studies pertaining to the applications of nanotechnology for CNS-based disorders were selected through algorithm application according to the recommendations of Page et al. [20,21]. Literature search was performed based on several scientific databases such as Google Scholar (http://www.scholar.google.co.in) (accessed on 10 November 2021), PubMed (http://www.ncbi.nlm.nih.gov/pubmed) (accessed on 10 November 2021), Elsevier (https://www.elsevier.com/en-in), ScienceDirect (http://www.sciencedirect.com) (accessed on 10 November 2021), Springer (http://www.springer.co.in) (accessed on 10 November 2021), and Scopus (http://www.scopus.com) (accessed on 10 November 2021). Only publications that had the full text available and book chapters restricted to the English language were reviewed, and figures were created using Biorender.com. (accessed on 10 November 2021).

## 3. Real Problem of Neurodegenerative Disease

**Figure 1:**
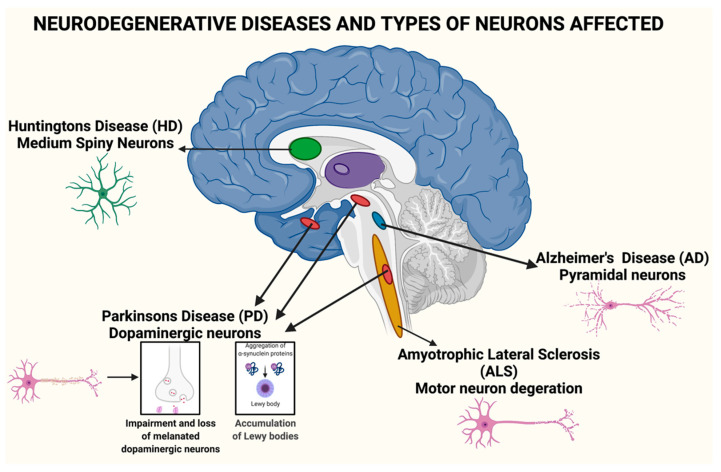
Neurodegenerative diseases and the types of neurons affected.

Numerous neurodegenerative sicknesses such as AD, PD, Huntington’s disease, and amyotrophic horizontal sclerosis (ALS), are connected to ageing. These neurodegenerative infections are critical social issues in numerous nations as the populace ages. The collection of misfolded and totalled proteins in the mind is an ordinary neurotic aggregate in these illnesses, as is shown in Figure 1. A developing assortment of proof demonstrates that poisonous protein conglomeration and neurodegenerative infections are connected. Revelatory endeavours for sickness-changing therapeutics have lately significantly expanded, because of a more noteworthy comprehension of the pathogenesis of these infections [22].

Neurodegenerative diseases are now the most common and debilitating illnesses afflicting humans. They have recently surpassed cancer, myocardial infraction (cardiovascular disorders), and stroke as the fourth leading cause of death. Neurodegenerative disorders disrupt a person’s reasoning, skilled gestures, emotions, cognitive actions, and memory, causing short- and long-term disabilities.

## 4. Role of Nanotechnology in Neurodegenerative Disorders

Neurodegenerative disorders are a collection of neurological illnesses that damage the brain or spinal cord’s neuronal structure and function. Because of the ageing population and the increasing prevalence of CNS disorders including Alzheimer’s, Parkinson’s, and strokes, the healthcare industry is faced with a tremendous issue. Early identification and treatment of CNS illnesses are still difficult despite advancements in our knowledge of their pathophysiology, and current medications are focused mostly on treating their symptoms. Due to unsatisfactory pharmacokinetics and unspecific targeting of new medications (such as proteins and peptides) that have been developed, many of these new treatments may not succeed because they may raise the risk of side effects. Biological barriers, such as the blood-brain barrier (BBB), are at the root of these difficulties. As a barrier between the CNS and peripheral circulation, it blocks the transfer of most chemicals from the blood to the brain [23]. In order to prevent chemicals, ions, and cells from crossing from the bloodstream into the central nervous system, BBB is made up of endothelial cells, astrocyte end-feet, and pericytes. The five primary ways by which chemicals traverse the BBB are diffusive transcellular lipoid bilayer membrane transport; transport carriers; specific receptor-mediated endocytosis and transcytosis; adsorptive transcytosis; and paracellular-tight junction transport. Toxins, infections, and inflammation are all prevented, and the CNS’s homeostasis is maintained, due to the presence of BBB [24]. Drug transport to the central nervous system (CNS) was further hindered by the BBB’s restrictive character. Furthermore, BBB malfunction has been reported in the majority of CNS illnesses, including Alzheimer’s, Parkinson’s, and strokes. Although it is unclear whether BBB failure is the primary cause of illness development, alterations in the transport system and enzymes are a significant contributor and exacerbator [24]. In CNS illnesses, the BBB impairment has major consequences for therapy. CNS illness patients may benefit from having an intact blood-brain barrier (BBB). In light of the fact that BBB malfunction plays a critical role in developing illness, this will undoubtedly be a divisive approach.

Nanotechnology, on the other hand, has advanced quickly in recent years and may offer significant benefits for the detection and treatment of CNS illnesses. Control or manipulation of nanometre-scale (one billionth of a metre) designed materials or devices is what is meant by the term “nanotechnology” [25]. Due to changes in the arrangement and spacing of surface atoms and molecules, nanomaterials exhibit markedly different characteristics than their macroscale counterparts [26].

There are several applications for biomarker discovery, treatments, and theranostics using nanomaterial-based technologies. To begin with, nanomaterials that have been changed may be utilised to identify and treat damaged cells and tissues at the molecular level. When a surface that has been modified with unique molecules, nanoengineered materials may also maintain medication release, boost bioavailability, distribute numerous agents, and preserve substances from degradation. Surface functionalization of nanomaterials may be used to target and infiltrate the BBB while also lengthening the nanomaterial’s blood half-life. For CNS illness diagnosis and treatment, nanomaterials are at the top of the list because of their unique features.

There are many promising advantages to be gained from using nanoparticles in the medical field, such as a high drug-loading capacity, which reduces the risk of chemical interactions or toxicity; a high surface area-to-volume ratio, which makes it easier to administer parenterally; the ability to use active and passive drug-targeting strategies; and sustained and continuous dosing options. Nanoparticle size, targeting properties, lipid, or water solubility and their respective hydrophobicity or hydrophilicity, chemical and physical stability, surface charge, and permeability, biodegradability, biocompatibility, cytotoxicity, drug release profile and antigenicity of the final product all play a role in the selection of the nanoparticle manufacturing materials.

NPs may be used for brain medication delivery because of their multifunctional and adaptable architectures. However, several factors must be considered before they may be used, including surface chemistry, hydrophobicity, shape, size, and charge, to name a few. Biocompatibility, decreased toxicity, and the capacity to bind and transport medications or therapies are all desirable features of an ideal NP. As long as it does not degrade quickly in vivo, it can pass across the BBB and regulate the release of medicines for extended periods of time. All of these characteristics result in NPs that are very effective at penetrating the BBB [27]. An investigation into site-specific drug delivery has resulted in a non-invasive distribution of the given dosage to specified areas, allowing for a precise and concentrated administration of the therapeutic dose at the site of the pharmacological activity. There are now non-invasive approaches, such as intranasal administration using drug modification to increase BBB permeability [28] as well as invasive ones, such as intraventricular or intracerebral injection or implantation, and infusion [29]. Disruption may also be effective in certain cases to cross the BBB [30]. The rupture of the BBB is often employed to improve the efficiency of medication transport to the brain. Mannitol, for example, is used to open the BBB to treat some CNS malignancies, and ultrasound is used to create temporary holes in the BBB [31]. Small therapeutic compounds (less than 1000 Da) have recently been shown to be able to pass across the BBB in Alzheimer’s disease (AD) and multiple sclerosis (MS) [32]. Physical and chemical features of NPs determine their path across the BBB and the methods by which they may cross it [33]. It is possible for NPs to penetrate the BBB and distribute medications in the sick brain when they are functionalized with a suitable ligand [34,35]. Endothelial cells may be crossed via transcytosis, allowing medications or drug-conjugated NPs to enter the central nervous system [36]; endothelial cells can be entered by endocytosis, allowing pharmaceuticals to cross the blood-brain barrier [37]. Researchers in neuropharmaceuticals are working to understand the processes of receptor-mediated and adsorptive transcytosis, as well as all the inherent physicochemical features of neuropharmeuticals. As a result, this might lead to new therapies that are more effective in crossing the blood-brain barrier (BBB) [38].

### 4.1. Techniques for Preparation of Nanoparticles and Nanocapsules

#### 4.1.1. Nanoprecipitation

Colloidal drug delivery devices may be integrated with active substances using the nanoprecipitation technique pioneered by Fessi et al. [39]. Some of the benefits of the generated nanoparticles include controlled release, targeted distribution, and better stability in biological fluids. As a result, you may expect minimal toxicity and moderate side effects. Preparing nanoparticles by nanoprecipitation requires a good solvent (usually an organic solvent such as ethanol, isopropanol, or acetone), whereas the particle is created using a non-solvent (such as water). Nanoprecipitation relies on both an organic and non-solvent phase, often referred to as aqueous phase, to guarantee that all initial components are completely soluble. The organic phase might include a low HLB surfactant and active compounds dissolved in an organic solvent or a mixture of organic solvents. The solubility of the active molecule in the solvent is one of the factors limiting the amount of medicine that can be loaded onto the particle. Particle formation and physical stability are made easier in the non-solvent phase by water-soluble stabilizing compounds [40]. There have been reports of particles devoid of stabilizing compounds. The amphiphilic nature of isoprenoid chains allows them to be linked to the active molecule in this situation. Nanoparticles form when the organic component is abruptly dumped or mixed with the aqueous phase. Particle size and polydispersity index are unaffected by nanoprecipitation, which is a reliable process. Instead, it seems that the features of the nanosized system are determined by parameters connected with the formulation used [40]. This might be linked to the procedures proposed for the production of nanoprecipitation particles. Nano and submicron particles can only be formed when particular polymer/solvent/nonsolvent ratios are used. As a consequence of the Gibbs–Marangoni effect (interfacial turbulence and thermal inequalities produced by mutual miscibility of the solvent and non-solvent and their varying interfacial tensions), mechanical mixing breaks down the organic phase into drops inside the aqueous phase [41]. Submicron-sized droplets of organic solvent diffuse away, causing the material that causes particle precipitation to become insoluble. It has also been suggested that the “ouzo effect”, which relies on the system’s chemical instability, might be a mechanism. In this situation, when aqueous and organic phases are joined, particle-forming molecules are supersaturated, allowing for the generation of “protoparticles” that follow the classic nucleation-and-growth process. Work circumstances must be created in order to enable the spontaneous generation of submicron or nanoscale particle sizes with the lowest polydispersity indices. As nanoprecipitation is difficult to standardize, polymer aggregation results in a wide and asymmetric particle size ranges. Low stabilizing agent concentrations and poor phase mixing are two obvious signs of polymer aggregates: a concentrated organic phase and a high organic phase ratio [40,42].

#### 4.1.2. Emulsification-Diffusion Method

So-called spontaneous emulsion diffusion describes a method for making biodegradable nanoparticles by dissolving a polymer in a mixture of water-immiscible and water-miscible solvents (such as dichloromethane) [43]. The quick dissolving of the miscible solvent causes the formation of a nanoemulsion when this solution is introduced to water. Immiscible liquid evaporates, forming nanoparticles. Nanoemulsions may be created by turbulence produced during solvent displacement, and there is no actual diffusion stage in the process of creating them. Solvent displacement and solvent evaporation are combined in this procedure, making it a hybrid. Due to the use of solely miscible solvents, the modified spontaneous emulsification solvent technique is obviously a method of solvent displacement [44].

The technique used to produce diffusion is a key variable in the emulsion diffusion approach. Because dilution water is added, low-solids dispersions are formed. So, armed with this information, it was suggested that the solvent (with a low boiling point) from the internal phase be extracted and then distilled directly into the exterior phase [45]. Due to the saturation of the continuous aqueous phase, its rapid displacement will prompt a free flux of the solvent globules to generate an anti-solvent medium, where the material will aggregate in the form of nanoparticles, in such a way that high solid concentrated dispersions (up to 30%) can be prepared from different materials such as polymers (e.g., poly(D,L-lactic acid), poly(!-caprolactone), and other materials. By using an emulsification-diffusion method, Yusuf et al. developed an emulsification solvent diffusion technique for coating with PS80 that reduced SOD1 levels and immobility, while raising acetylcholinesterase levels in Piperine-SLNs produced with Piperine. In addition, histological investigation revealed decreased plaques and tangles [46].

#### 4.1.3. Double Emulsion Technique

The most popular techniques for evaporating solvent from emulsions are the single- and double-emulsion procedures. This process uses an organic phase that has been emulsified in an aqueous medium with surfactants or stabilisers before the solvent is evaporated to create the NP [47]. Double emulsion solvent technique uses a three-phase process, which is different from the single emulsion solvent method, which uses just two phases. Shear stress during the homogenization process is the fundamental downside of these two methods, which results in poor protein encapsulation effectiveness. The nanoprecipitation approach may help overcome this issue. Dropwise addition to the aqueous media of the organic solvent containing PLGA results in the fast diffusion of the miscible solvent, resulting in the creation of the NPs layer-by-layer method [48].

Sukhorukov et al. devised the approach for the production of vesicular particles. Adsorbed polymer layers may be applied to a colloidal template using either the polymer solution and washing or the addition of a miscible solvent to reduce polymer solubility. Chitosan, heparin, polylysine, protamine sulfurate, gelatine, and dextran sulphate are examples of common polymers that may have polyanionic or polycationic characteristics [49,50].

### 4.2. Green Method of Synthesis of Nanoparticles

Research and development in materials science and technology is entering a new phase in which “green synthesis” methodologies and technologies are becoming more popular. A major benefit to environmentally friendly synthesis of materials and nanomaterials will be improved by regulation, control, clean-up, and remediation of the production process.

‘Green synthesis’ is necessary in order to prevent the generation of undesirable or dangerous by-products via the development of dependable, long-lasting, and environmentally friendly synthesis methods. In order to attain this purpose, it is critical to make use of appropriate solvent systems and natural resources. The use of metallic nanoparticles in the green synthesis of biological materials has been widely accepted (e.g., bacteria, fungi, algae, and plant extracts). Using plant extracts to synthesise metal and metal oxide nanoparticles is one of the more straightforward green synthesis techniques accessible, especially when compared to bacteria- and fungus-mediated approaches. Biogenic nanoparticles are a grouping of these compounds.

Various reaction factors, such as solvent, temperature, pressure, and pH conditions, influence green synthesis methods based on biological precursors (acidic, basic, or neutral). Many plant extracts, particularly those from leaves, include potent phytochemicals, such as ketones, aldehydes, flavonoids, amides and terpenoids, carboxylic acids, phenols and ascorbic acids, that may be used to synthesize metal and metal oxide nanoparticles [51,52].

## 5. Role of Phyto-Nanomedicine on Neurodegenerative Diseases Treatment

Neurodegenerative Diseases (ND) are basically disorders related to age and are prevailing rapidly worldwide due to the increased elderly population in recent years. The actual problem however is not the increasing prevalence but the lack of effective treatment. As per published evidence from the past, NDs were discovered in the early 1900s, however, presently, no effective treatments for these disorders are found in modern society. Integrative medicine can sometimes delay the progression of these diseases or exhibit protective effects. It is believed by scientists that with the development of integrative medicine and modern science, the former will progressively take a more and more significant role in the treatment of ND [52,53].

A series of synthetic drugs have shown positive results for the treatment of some common NDs including Autism, PD, AD, and other chronic illnesses. The use of synthetic drugs is associated with many side effects, which make them inappropriate for regular treatment. Considering the adverse effects of these synthetic drugs, scientists have made a soft turn towards the utilization of phytochemicals, as they have minimal side effects. The antioxidative, anticholinesterase, anti-inflammatory, and anti-amyloid properties of phytochemicals makes phytochemicals a promising therapeutic agent [54,55].

In consideration that current therapeutics seem to be inadequate treatment for the above-mentioned disorders, scientists are exploring their options with plant-based drugs using nanotechnological approaches. Nanotheranostics is one such approach which is gaining wide attractions from the scientific community at a global level for the treatment of ND. It uses nanoparticles for the simultaneous diagnosis as well as for treatment. Research findings of Tripathi et al. [56] reveal that this treatment has been receiving significance in the medical field because it is extremely aggressive and specifically targets the affected area. Moreover, alterations with respect to type of disease and personalization based on the needs of the patient can be met, which further increases the applicability of the approach [57]. A novel nanotheranostic system has been developed by chemical engineers, which uses adjustable light that activates nanoparticles and opens new applicability in the field [58].

In the subsequent sections we will discuss in detail the various types of plant-based medicines that are utilized in ND treatments along with the conventional approaches and recent trends in the plant-derived nanomedicine discovered so far.

### 5.1. Types of Phyto-Medicines Available for Treatment

▪ Acorus *calamus*

Acorus *calamus,* also commonly referred to as sweet flag, belongs to the family Acoraceae and acts as a nervous system rejuvenator and has beneficial effects on the brain through memory enhancement, learning behaviour, and performance modification. Acorus *calamus* consists of a majority of α-and β-asarone while β-asarone brings about the suppression of beta-amyloid-induced neuronal apoptosis observed in the hippocampus through the downregulation of Bcl-w and Bcl-2, which further causes the activation of caspase-3 and phosphorylation of c-Jun N-terminal kinase (JNK). Apart from the above-mentioned benefits parts, of Acorus *calamus* also showed an inhibitory result on AChE with an IC50 value of 188 μg/mL. It has also shown positive results by improving the dopaminergic nerve function, and thus acts as neuroprotective for PD [59].

▪ Allium *sativum*

Allium *sativum,* commonly known as garlic, belongs to the family Amaryllidaceae and has been used since ancient times for its medicinal properties. It was used for cardiovascular disease as well as for the prevention and treatment of other metabolic diseases such as hyperlipidemia, atherosclerosis, thrombosis, dementia, hypertension, diabetes, and cancer. S-allyl cysteine (SAC), one of the major constituents found in aged garlic extract (AGE), has been extensively studied. SAC has indirect and direct antioxidant activity. Besides bringing about a decrease in lipid peroxidation and DNA fragmentation, it also causes a reduction in oxidation and nitration. In the Parkinsonian models, namely 1-methyl-4-phenyl pyridinium (MPP) and 6-hydroxydopamine (6-OHDA), SAC causes a protection of dopamine levels, prevents oxidative damage and causes peroxidation of lipids. [60].

▪ Bacopa monnieri

Bacopa *monnieri* (Linn), popularly called “Brahmi” (family Scrophulariaceae), is a herb found in tropical countries such as India. Steroidal bacosides (A and B) and saponins are the main active compounds present in Bacopa *monnieri*. These active compounds are responsible for enhancing memory and learning. Other constituents include bacopa saponins F, E, and D, flavonoids, phytosterols, and alkaloids. Superoxide SOD, CAT, GPx, and glutathione reductase (GSR) activity is enhanced by Bacoside A. Therefore, the levels of glutathione in the brain are upregulated significantly. Bacoside A inhibits lipid peroxidation by modifying the activity of enzymes such as Hsp 70 and cytochrome P450 in the brain. It also improves the activities of adenosine triphosphatases (ATPases), maintains ionic equilibrium, and restores level of selenium and zinc in the brain. The reduction in aggregation of alpha-synuclein protein by Bacopa *monnieri* was also found by researchers [61].

▪ Centella *asiatica*

Centella *asiatica* belonging to the Apiaceae (Umbelliferae) family and has been demonstrated to possess a neuroprotective property. Since ancient times, it was part of the Ayurvedic system as an alternative medicine for bringing about memory improvement. Centella *asiatica* decreases Aβ deposition in the brain, exhibits potent antioxidant activity and can scavenge free radicals, causes a reduction in ferric ions, and brings about a restoration of GSH levels through an upregulation of glutathione-S-transferase activity. Chen et al. [62] carried out a study which suggested that Centella *asiatica* ethanolic extract causes the suppression of Aβ-induced neurotoxicity through the enhancement of the antioxidative-based defence system in IMR32 and PC12 differentiated cells. Amelioration of decrease in AChE activity due to colchicine-induction, neuronal damage through induction of asiaticoside occurring through nitric oxide inhibition, also reflects the neuroprotective effect of Centella *asiatica* [63].

▪ Curcuma *longa*

Turmeric, derived from the plant Curcuma *longa,* belonging to the Zingiberaceae family, is a gold-coloured spice and has been used traditionally as medicine for a variety of diseases [64,65]. Curcumin, turmeric’s principal constituent, has several known neuroprotective actions. In patients afflicted with AD, studies have shown that curcumin potentially binds to Aβ peptides, preventing aggregate formation of new amyloid deposits as well as promoting the de-aggregation of the existing amyloid deposits. Analogues of curcumin, namely desmethoxycurcumin and bis-desmethoxycurcumin, have also shown protective ability against Aβ-induced oxidative stress. Moreover, curcumin causes the inhibition of Aβ oligomerization and formation of fibril, causes a macrophage enhancement of Aβ uptake, and inhibits the A beta-heme complex peroxidase activity. Other components of turmeric such as curcuminoids and polyphenolic compounds attenuate mitochondrial dysfunction, inflammatory response induction, and oxidative stress in the presence of inflammatory cytokines such as iNOS and COX-2. Curcuminoids can also bind to Aβ plaques, causing inhibition of amyloid accumulation and its aggregation in the brain [63].

▪ Celastrus *paniculatus* Wild

Celastrus *paniculatus* Wild, commonly called as Jyotishmati, belongs to the Celastraceae family. Traditionally, it was administered as a powerful appetite stimulant, brain tonic, and emetic. Phytochemical studies show the presence of a sesquiterpene, evoninoate, alkaloids paniculatine A and B, wifornine F celapagine, celapanine, celastrine, celapanigine, polyalcohols such as malkanginnol, paniculate diolmalangunin, and malkanguniol, triterpenoids, and sterols such as β-amyrin and β-sitosterol. Scientific studies suggested that Celastrus *paniculatus* water extract modulated the glutamate receptor function that protects neurons against toxicity produced by the glutamate and resulted in the improvement in memory and learning. It also causes a noteworthy decrease in the MDA level in the brain, which is an important marker of oxidative damage, with simultaneously significantly increases in levels of glutathione and CAT; two endogenous antioxidants in the brain [63]. Jakka et al. [66] investigated research that explained the neurotrophic potential in Celastrus *paniculata* Wild whole plant methanolic extract (CPPME). A significant decrease was also seen in AChE and enhanced neurotrophic activity, which ultimately improved spatial memory formation in scopolamine-induced amnesia.

▪ Coriandrum *sativum L*

Coriandrum *sativum L*., also referred to as dhanya, belongs to the family Apiaceae [30]. The major phytochemicals present include flavonoids such as quercetin 3-glucoronide, and polyphenolics such as protocatechinic acid, glycitin, and caffeic acid. In seeds, the flavonoid content was reported at a concentration of 12.6 quercetin equivalents/kg and the polyphenolic concentration was reported at 12.2 gallic acid equivalents/kg [67,68]. A study reported that the Coriandrum *sativum* extract caused an increase in total protein concentration and enzyme levels of CAT, SOD, GSH, and reduced the size of cerebral infarct, calcium levels, and lipid peroxidation (LPO) in the experimental rat. Scopolamine- and diazepam-induced memory deficits were also decreased by Coriandrum *sativum* leaves. The leaves also show an antioxidant property, having radical scavenging activity of DPPH with lipoxygenase inhibition and phospholipid peroxidation inhibition activity, which contribute to its memory enhancement effect [69].

▪ Galanthus *nivalis*

Galanthus *nivalis,* referred to commonly as snowdrop, belongs to the family Amaryllidaceae. Galantamine is the major constituent found in the bulbs and flowers of Galanthus *nivalis* and is a tertiary iso-quinoline alkaloid. The neuroprotective effect exerted by Galantamine is associated with a dual action. The drug is a competitive and selective AChE inhibitor and can stimulate nicotinic receptors, which further enhance cognition and memory [70].

▪ Ginkgo *biloba*

Gingko *biloba* belongs to the Ginkgoaceae family and is considered a ‘living fossil’. Its extract majorly consists of flavonoids, whose fraction accounts to 24%. It mainly comprises of flavanols such as, isorhamnetin, quercetin, kaempferol, and terpene lactones, which account to a concentration of 6% and further comprise diterpenic lactones such as ginkgolides A, B, C, J and M, and a sesquiterpene tri lactone-bilobalide. The extract exhibits neuroprotection by several mechanisms such as membrane lipid peroxidation inhibition, anti-inflammatory activity, as well as through direct inhibition of amyloid-b aggregation and anti-apoptotic activities. The flavonoid fraction of Ginkgo *biloba* (G. *biloba*) extract has a free-radical scavenging property as well as anti-oxidative effects, while bilobalide has been associated with a decrease in damage caused due to excitotoxicity and global brain ischemia-induced death of neuronal cells. Extract of G. *biloba* in the brain significantly inhibits the AChE activity, which indicates that the level of acetylcholine has increased considerably. Flavonoids alter several biological processes through interaction with signalling pathways, effects on protein neuronal expression, which is imperative for plasticity and repair of the synapse, variation in cerebral blood flow, and neuropathological inhibition of processes in certain cortical regions. A study by Dash SK presented that the G. *biloba* extract causes a decline in cortical Aβ levels through a reduction in cholesterol levels as free and circulating cholesterols affect amyloid genesis [71].

▪ Glycyrrhiza *glabra*

Glycyrrhiza *glabra,* widely referred to as Yashti-madhuh or liquorice, belongs to the Leguminosae family. Its major constituent is a flavonoid called Glabridin, which possesses multiple pharmacological activities such as anticancer, antiviral, anti-ulcer antioxidant, anti-diabetic, immunomodulatory activity, anti-inflammatory activity, antimicrobial activity, and anticonvulsant activity. Liquorice significantly increased learning and memory, however, research has indicated that its consumption improves general intelligence rather than short-term memory. In the brain, glabridin decreases the MDA level, and increases the superoxide dismutase level while reducing glutathione levels. A study suggested that G. *glabra* administration restored the decreased concentration of dopamine and glutamate in the brain and decreased activity of AChE [72].

▪ Hypericum *perforatum*

Hypericum *perforatum* is also known as millepertuis or hypericum. It belongs to the family Hypericaceae. Although it is found worldwide, it is mainly native to Europe, northern Africa, and western Asia. The main active component of H. *perforatum* is Hyperoside. Hypericin, kaempferol, biapigenin, and quercetin comprise the other constituents. The extract of H. *perforatum* has been reported to behave as a protector against NADPH-dependent (enzymatic) and Fe^2+^ and ascorbate-dependent (non-enzymatic) peroxidation of lipids in the cortical region of the brain. The extract also protects brain cells from cytotoxicity brought about by glutamate through the reduction of glutathione loss, overload of calcium, and cell death mediated through ROS. The ethanolic extract of H. *perforatum* may also bring about an improvement of microglial viability through the reduction of toxicity by amyloid-beta in AD. Hypericum *perforatum* inhibits AChE and MDA formation in the brain and increases the concentration of SOD, GPx and CAT. Therefore, H. *perforatum* also has the capability to bind to iron ions, has scavenging activity for hydroxyl radicals, and behaves as an antioxidant [73].

▪ Lycopodium *serratum*

Lycopodium is also referred to as ground pines or creeping cedar. It belongs to the family Lycopodiaceae, which comprises a family of fern-allies. Its leaves contain a single, unbranched vascular strand and are microphylls. The major constituent is huperzine A, which is a potential therapeutic agent by researchers for treating AD. The alkaloid, which has been isolated from Lycopodium *serratum,* has been used for treating inflammation, fever, and blood disorders for many decades. It acts as a highly reversible, potent, and selective inhibitor of AChE and is comparable to the potency observed by galantamine, physostigmine, tacrine, and donepezil. Huperzine A is considered to be a strong candidate for therapy in AD. It has been found that huperzine A causes a noteworthy upregulation in AChE levels in rat brains and is associated with protective effects such as amyloid precursor protein metabolism regulation, oxidative stress protection mediated by Aβ, apoptosis, dysfunction of mitochondria, and anti-inflammation [74].

▪ Melissa officinalis

Melissa *officinalis* L. (Lamiaceae) leaves are commonly referred to as lemon balm and have been used traditionally for spasmolytic and nerve calming effects. The leaves produce a calming and soothing effect through interaction with the GABA_A_ benzodiazepine receptor. Its extracts contain flavonoids, namely quercitrin, apigenin, luteolin along with phenolic acids. The derivatives of these products inhibit enzymes such as monoamine oxidases (MAO) and AChE, which scavenge these free radicals and prevent apoptosis. Enzymatic inhibition of the above-mentioned enzymes leads to alleviation of depression symptoms. Research also suggests that Melissa *officinalis* produces protective effects in the PC12 cell line and protects neurons from oxidative stress [75].

▪ Ocimum *sanctum*

Ocimum *sanctum* is commonly referred to as ‘Tulsi’ in Hindi and in English, ‘Holy Basil’. It belongs to the Labiatae family. The plant is reported to contain glycosides, alkaloids, saponins, tannins, vitamin C, citric acid, tartaric acid, and maleic acid. Research conducted by Kusindarta et al. [76] indicated that an ethanolic extract derived from the leaves of Ocimum *sanctum* may stimulate and restore choline acetyltransferase expression in human ageing cerebral microvascular endothelial cells and could provide nerve protection and increased production of Ach, which may enhance memory and cognitive ability. Scientific studies reveal that the hydro-alcoholic extract of Ocimum *sanctum* exhibits strong antioxidant activity against DPPH and hydroxyl radicals, which possibly occur due to the presence of a high number of polyphenols and flavonoids. It inhibits peroxidation of lipids, ROS generation, damage to DNA, and depolarization of membranes. It also decreases the enzymatic leakage of lactate dehydrogenase, preserves cellular morphology, restores superoxide dismutase, and catalyses enzyme levels, thereby preventing neuronal damage [77].

▪ Panax Ginseng

Ginseng belongs to the Araliaceae family and is prominently found in north-east Asia. It is used world-wide for boosting energy. Ginseng may provide neuroprotection against neuronal degradation through various mechanisms such as production of a reduction in the β-amyloid deposition or glutamate-induced excitotoxicity in a dose-dependent manner, thereby preventing apoptosis and neuronal death, improving routine in a passive-avoidance learning paradigm, and protecting neurons possibly through its potential to suppress cellular AChE activity and enhance cholinergic metabolism [78].

▪ Rosmarinus *officinalis*

Rosemary, commonly known as Satapatrika, belongs to the Lamiaceae family. It contains many essential oils such as eugenol, carvacrol, oleanolic acid, and ursolic acid, and thymol constituent’s antioxidants such as ferulic acid and carnosic acid, which can be used against cyanide-induced damage in brain. A neuroprotective effect is also seen, which can be cultured and human-induced, as has been observed in rodents, cell-derived neurons, and pluripotent stem cells in vivo and in vitro in various parts of the brain in non-Swiss albino mouse models. It also possesses cytoprotective, anti-apoptotic, and anti-inflammatory activities that also add on to its neuroprotective mechanism [79].

▪ Salvia officinalis

Salvia *officinalis* belongs to the Lamiaceae family, and is well-known and reputed for improving memory, as it has been traditionally used as a memory enhancing agent. Carnosic acid and rosmarinic acid are the active ingredients found in S. *officinalis*. These components are known to have potential pharmacological activity such as antioxidant and anti-inflammatory properties as well as a low AChE inhibitory effect. It inhibits ROS formation, peroxidation of lipids, fragmentation of DNA, activation of caspase-3, and hyperphosphorylation of protein tau. Clinical evidence that has been obtained may help to prevent or reduce the symptoms of dementia. A small pilot trial involving the oral administration of S. *officinalis* essential oil to 11 patients that possessed mild-to-moderate symptoms of AD improved cognitive function significantly [80].

▪ Terminalia *chebula*

Terminalia *chebula* (T. *chebula*), also known as “King of Medicines” in Tibet, belongs to Combretaceae family. It has been widely used as traditional medicine in Ayurveda, Siddha, Unani, and Homeopathy. It contains compounds such as arjungenin, triterpene sarjunglucoside 1, and the tannins, chebulosides 1 and 2, chebulic acid, chebulinic acid, tannic acid, ellagic acid, 2,4-chebulyi–β-D-glucopyranose, gallic acid, ethyl gallate, punicalaginterflavin A, and terchebin. It also has presence of flavonoids such as rutins, luteolin, and quercetin. A study reported that T. *chebula* exhibits anxiolytic activity and is equivalent to standard drug diazepam. T. *chebula* has good pharmacological activities relevant to dementia therapy and possesses antioxidant activity comparable to radical scavengers such as quercetin, reflecting 95% activity, and showing an inhibitory concentration (IC50) value of 2.2 μg/mL. T. *chebula* fruit extract also shows protective effect neuronal cells against ischemia, reduces least production, and stimulates microglia cells death rate by lipopolysaccharide [81].

▪ Tinospora *cordifolia*

Tinospora *cordifolia* (T. *cordifolia*) belongs to the Menispermaceae family, which is commonly known as giloe. Chemical constituents extracted from the plant are alkaloids, diterpenoid lactones, steroids, glycosides, and aliphatic acids. T. *cordifolia* holds a memory increasing property, which is due to immune-stimulation and increased synthesis of acetylcholine. T. *cordifolia* exhibits the property of scavenging free radical activity against ROS and reactive species of nitrogen, which have been studied through electron paramagnetic resonance spectroscopy. It increases the concentration of glutathione and the expression of the gamma-glutamyl-cysteine ligase and superoxide dismutase of copper-zinc genes, which play a major role in neuronal injury during hypoxia and ischemia. In addition, T. *cordifolia* significantly decreases the mRNA expressions of iNOS. T. *cordifolia* also increases the dopamine level of the brain. Thus, T. *cordifolia* has shown to prevent neurodegenerative changes and enhance cognition, learning, and memory [82].

▪ Withania *somnifera*

Withania *somnifera* belongs to the family Solanaceae and is popularly known as Ashwagandha or Indian ginseng. The major constituents of Ashwagandha root are two withaferin A, withanolide D, and withanolides. Active glyco-withanolides of Withania *somnifera* have a significant antioxidant function, which is accomplished by elevating the activities of SOD, CAT, and GPx. It is also reported that Ashwagandha also works as a nerve tonic that boosts energy and rejuvenates the cells. According to Rajasankar et al. [83], treatment of PD mice with an oral dose of Withania *somnifera* root extract (0.1 g/kg body weight) for 1 week or 4 weeks enhanced homo-vanillic acid and dopamine, 3,4-dihydroxy phenyl acetic acid levels in the corpus striatum. Furthermore, the report suggested that Withania *somnifera* treatment enhances the anti-apoptotic proteins level such as Bcl-2 and depreciates the pro-apoptotic protein level such as Bax in the Maneb–Paraquat-induced dopaminergic neurodegeneration model of PD. Ashwagandha extract has shown to prevent lipid peroxidation and increase antioxidant activity by increasing the free-radical slinking enzymes levels in the brain [63].

▪ Zizyphus Jujube

Jujube fruits are used in Korean and Chinese traditional medicine to reduce anxiety and strengthen the stomach and gastrointestinal system. Jujube seeds comprise large amounts of flavonoid, phenyl glycosides, terpenoid, and alkaloid compounds mucilage, citric acid, malic acid, sugar, organic minerals, vitamin C, and protein. The herb exerts inhibition activity against the release of histamine, cyclooxygenase I and II, and AChE inhibitory activity. Flavonoids present antioxidant properties [63]. A compound known as cis-9-octadecenamide (oleamide) extracted from jujube is reported to bring about an upregulation of acetylcholine transferase to 34.1% in the in vitro models, which leads to the increase in acetylcholine level and improves mild-to-moderate cognitive functions, motor coordination, behavioural disorders, learning, and memory [84].

### 5.2. Conventional Approach

As a harsh reality in ND and As, the only hope is symptomatic treatment. The symptomatic treatment therapies rely on slowing down symptoms without addressing the cause and cure of the disease. Apart from symptomatic treatment, the other strategy that is being employed currently is disease modifying-based treatment. Inhibitors of anti-cholinesterase are used as symptomatic treatment, while anti-inflammatory agents and antioxidants are used for disease modifying treatment [85]. Current therapies for neurodegenerative and neurological disorders usually are involved in managing symptoms instead of providing significant activity against disease progression. For example, the symptoms of HD are controlled using 75–200 mg/day of tetrabenazine to alleviate involuntary movement (chorea). However, as it acts as a vesicular monoamine transporter inhibitor (VMAT), it causes an interference with both 5-HT (5-hydroxytryptamine) and dopamine (DA) degradation, and, as a result, patients can show neuropsychiatric-based symptoms along with various other side effects [86]. Various other first-line treatments such as L-Dopa in PD often cause a multitude of side effects and do not delay progression of the disease. Another example is that of cholinesterase inhibitors such as Donepezil, which is minimally effective in providing improvement in cognition required for AD treatment. The various conventional strategies used currently in the treatment of the two most common NDs, AD and PD, are summarized in detail in Table 1 [87].

In view of the above information, there is an essential requirement for the development of novel therapeutics that possess lesser or tolerable side effects to overcome these disease states, which can further improve the quality of life of the ageing population. Conventional drug delivery strategies, in general, fail to cross the BBB and are therefore less efficacious in terms of treatment [88].

## 6. Recent Trends of Phyto-Neuro Medicine

The latest advancements in the domains of green chemistry and nanotechnology are promising and indicate significant potential in the advancement of biomedical sciences from a theranostic perspective [89,90]. However, this potential, so far, has not been utilized for the development of therapeutics for NDs such as AD and PD. The utilization of chemically synthesized molecules poses limitations such as toxicity and cost [89,91,92]. In addition, a study reported that some chemicals that are used for the chemical synthesis of NPs have the propensity to remain attached on the NP surface and as a result, could not be applied biomedically. Therefore, focus has moved towards the development of materials using green chemistry and a green process [93]. Traditionally, the utilization of the green chemistry-based approach is based on the use of medicinal plants or phytochemicals in their pure form that possess medicinal properties, as these phytochemicals provide chelation and stability to the NPs [90].

Recently, Suganthy et al. [94] reported the neuroprotection offered by biogenic gold NPs based on Terminalia arjuna. The results obtained showed significant biocompatibility of biogenic gold NPs as well as significant neuroprotection. These particles successfully inhibited the AChE, caused a reduction in the Aβ fibrillation process, and at low concentrations caused a destabilization of mature fibrils. Trehalose functionalization of synthesized gold NPs brought about a significant improvement in the inhibition of protein aggregation along with the disintegration of mature fibrils, and possesses the potential for application in photothermal therapeutics [56]. Functionalization of gold NPs with anti-amyloidogenic molecules can be considered as a promising strategy for ensuring improvement in the neuroprotective nature. Another strategy involves the use of biogenic platinum NPs biosynthesized through utilization of Bacopa *monnieri* as a neuroprotective agent. Functionalization of NPs using phytochemicals has yielded significant results. Recently, functionalization of selenium NPs using polyphenols was achieved, where in nanoscale selenium nanoparticles were coated with EGCG, which is a polyphenol present in tea. EGCG does possess neuroprotective effects as well as inhibitory actions against a multitude of proteins that are amyloid-forming such as amyloid beta, transthyretin, α-synuclein, and huntingtin, which are involved in AD disease progression. These particles were further coated with Tet-1, which is a protein that has strong affinity to neurons. The use of curcumin and its derivatives in a similar manner has led to therapies that show promising results in patients with AD. Benzothiazolinone in conjugation with curcumin has a strong binding affinity to amyloid and therefore can be used for targeted delivery of curcumin or various other natural products that can help in the treatment of AD [95].

Because of its significant antioxidant and anti-inflammatory effects, Yusuf et al. created PLGA NPs loaded with thymoquinone (TQ) to operate on this aspect in animal models [96]. Streptozotocin (SZT)-treated male albino mice were treated with TQ-loaded PLGA NPs coated with polysorbate 80, which mimics AD oxidative stress by decreasing Streptozotocin (SOD) activity (P-80-TQN). With an average particle size of 226 nm and a zweta-potential of 45.6 mV, the single-emulsion solvent evaporation technique was used to create these nanoparticles. Initial bursts of TQ release were seen after 2 h, followed by a prolonged sustained release (stabilised dipole-dipole interactions taking place between TQ and PLGA components). Endocytosis via LDL receptors allowed the P-80-TQ NPs to traverse the BBB (mediated by the polysorbate coating). These systems had a considerable impact on SOD activity (increase) from the seventh to the 28th day after arrival on site. Further proving their positive impact, a study on animals and cognition (the “Despair test”) was conducted at the same time.

Uncaria species have yielded the spirocyclic alkaloid rhynchophylline (RIN), which has been shown to have a variety of pharmacological effects, including neuroprotection. RIN suppresses soluble A-induced hyperexcitability of hippocampus neurons in the event of Alzheimer’s disease. In 2020, Xu et al. released the first research on the design and development of brain-targeting treatment for Alzheimer’s disease via RIN injection [97]. Methoxy Polyethylene Glycol NPs coated with Tween 80 were synthesised using the nanoprecipitation process to improve RIN’s pharmacological activity and target specificity. T80-coated RIN-loaded PLGA NPs showed no haemolysis, indicating that these NP solutions might be safely used. In confocal laser scanning microscopy, internalisation into bEnd.3 cells was demonstrated using DiD fluorophore-loaded PLGA NPs; T80-coated NPs showed more penetration. An in vitro BBB model using bEnd.3 cells was constructed to evaluate the crossing of such nano-systems and indicated their greater transport relative to free RIN or uncoated RIN laden NPs. Utilizing healthy C57BL/6 mice, the advantages of using T80 to target the brain were subsequently shown. Ultimately, incubation with T80 RIN NPs increased the survival rate of PC12 cells damaged by A25–35 while also reducing cell death. RIN neuroprotective effects were unaffected by the encapsulation of PLGA nanoparticles, as shown by these findings.

In totality, phyto-nanomedicines are promising as compared to currently marketed therapies for neurodegenerative disorders. Phytochemical derived nanomedicine could prospectively in future be used for neurodegenerative disorders due to their multitude of properties such as anti-inflammatory, antioxidative, and anticholinesterase activities. Further research, however, is necessary to investigate the complete neuroprotective ability of these compounds, to understand the mechanisms by which they exert protective effects, as well as determining whether combination therapy could be synergistic as neuroprotectants [85].

Undoubtedly, phyto-nanomedicines offer a great hope in developing treatment strategies against ND because of their fewer side effects and better target specificity. However, there are still a few limitations which must be considered. A limitation of the utilization of making use of the nanotheranostics for the treatment of NDs was identified by Kumar et al. in 2020. They found that due to variations in neural functioning, characteristics, and genome, a particular single approach cannot be used for all patients for the treatment of a particular disease. Another study by Kumar et al. [88] showed that this technique was proving to be ineffective as the NPs absorption time used in the technique was very low and if the injection is not performed adequately, there is a possibility of NP absorption in the blood or other body parts instead of the targeted area. Another constraint that was identified was that the treatment cannot be monitored in an effective and efficient manner, which poses a major challenge for the practitioners as they would be unable to track the progress and impacts of the treatment. In addition, the treatment and technique, besides being unproductive, is also extremely costly [88].

### 6.1. Protein-Based Nanoparticles

Because of their low toxicity and biodegradability, biopolymer-based nanoparticles, such as protein nanoparticles, have recently been actively employed as medicinal and functional tools [98]. Proteins are versatile building blocks for nanoparticles because of their specific roles in biology and the industrial industry. Endocytic transport of protein nanoparticles is possible because of their tiny size. As a drug delivery strategy, protein nanoparticles offer a number of benefits, including biodegradability, stability, surface modification, simplicity of particle size control, and less concerns related with toxicity issues, such as immunogenicity. Protecting the medication against enzymatic breakdown and renal clearance may increase its stability, activity, and half-life in particular. Additionally, protein nanoparticles may be employed in a number of targeted therapeutics, including cancer therapy, tumour treatment, and vaccinations, because of their non-antigenic properties. When protein nanoparticles are embedded in biodegradable polymers, they may be released over a long period of time. It is the primary goal of nanoparticle design to manage particle size and surface area so that nanoparticles containing the requisite quantity of pharmaceuticals may demonstrate desirable pharmacological activity by discharging active substances to produce part-specific action.

In silk fibres, the fibroin protein accounts for between 65 and 85 percent of the total protein content [99]. Degumming with Na_2_CO_3_ removes exterior sericin from silk produced by the Bombyx mori silkworm, a technique often used to extract fibroin [100]. As a result of its excellent mechanical strength, flexibility, low immunogenicity, biodegradability, and biocompatibility, fibroin has become a popular choice for the creation of nanoparticles. Fibroin nanoparticles’ zeta potential is negatively charged. When a positively charged polymer such as PEI or chitosan is applied to the surface, it serves as a crosslinking agent that transforms the surface into a positive charge. The average size, size distribution, surface zeta potential, drug encapsulation, release profile, and particle formation stability of FNP may be affected by a variety of parameters, including fibroin molecular weight (MW), crystallinity, encapsulated drug characteristics, and production circumstances. Much research has been undertaken to distribute and use fibroin nanoparticles as a medication delivery method because they can overcome the drawbacks of low-molecular-weight medicines. Drug solubility and stability are enhanced, drug degradation is inhibited, and toxicity is reduced in all fibroin nanoparticles loaded with small molecule pharmaceuticals, making them better for drug therapy.

Approximately 585 amino acids make up HSA, which has a molecular weight of 66 kDa [101]. HSA is mostly present in the bloodstream. Subunits A and B may be found in each of the three major components of the HSA. Sudlow’s sites I and II, found in subunits IIA and IIIA of the HSA, are the primary binding sites [102]. When HSA is used as a carrier for other compounds, it helps hydrophobic molecules become more easily soluble in blood. A variety of chemicals may be delivered to particular tissues in the body via HSA. The pH (stabilised in the pH range of 4 to 9) and temperature (may be heated at 60 °C for up to 10 h) of HSA are also highly stable, as they are organic solvents [103]. Because of its biodegradability, non-toxicity, and non-immunogenicity, as well as its high solubility, it has the added benefit of being derived from biological sources. In investigations on protein binding and targeted medication administration, bovine and HSAs are often used because of these benefits. Due to HSA’s strong affinity for diverse medications, a matrix of HSA nanoparticles may efficiently incorporate these molecules.

Table 2 describes the outcomes obtained following administration of drug-entrapped NPs for neurological disorders.

### 6.2. Polymeric Nanoparticles

Polymeric nanoparticles are colloidal particles that have a medication encapsulated inside a biodegradable and biocompatible polymer carrier, ranging in size from 1 to 1000 nm [100,113]. Polylactide, polylactide polyglycolide copolymers, and polyacrylates are some of the most widely used polymers. Among them, Lactide glycolide copolymer has been widely studied. There are several natural polymers to choose from, including alginate, albumin, and chitosan. Various research has been ongoing for many years to establish the efficacy of curcumin (Indian solid gold) in a variety of disorders, including cancer. It was shown that curcumin was able to reduce Amyloid beta deposition and tau phosphorylation in an animal study on AD.

In addition, it increases the multiplication of neural stem cells and hippocampus neurogenesis [114]. As Curcumin has a weak water solubility, the nanoparticle format of Curcumin is thought to boost its ability to target neurons in Alzheimer’s disease (AD). Curcumin nanoparticles Tet-1-targeted PLGA-coated curcumin were tested for the treatment of Alzheimer’s disease by suppressing amyloid and antioxidant activities [107]. Nanoparticles having a zeta potential of 230 to 220 mV and an average size of 150–200 nm were manufactured using the solvent evaporation technique. These nanoparticles are entirely soluble in water, and they also have the ability to glow. Studies on the viability of cells have shown that these nanoparticles are not harmful to cells. According to fluorescence data, the uptake of nanoparticles targeted with Tet-1 peptide in GI-1 glioma cells in an in vitro uptake assay was significantly increased compared to the non-targeted nanoparticles.

#### 6.2.1. Liposomes-Based Drug Delivery Systems

Liposome transport mechanisms across the blood-brain barrier are still at the budding stage. Electrostatic interactions are the method by which the negative charges on BBB and cationic liposomal drug delivery systems induce cell internalisation through absorption mechanism [115]. It is possible to traverse the BBB by attaching glucose and GSH to liposomes as nutrients. Because of the therapeutic potential of receptor-mediated transcytosis, which has the ability to connect specific ligands to a wide variety of BBB receptors [116]. Using nanoliposomes that have been double-functionalized with curcumin and HIV TAT peptide, A peptide affinity is increased and BBB bridging is improved [117]. Endocytosis and micropinocytosis were the two most often cited mechanisms for TAT uptake. A thiol-maleimide reaction covalently links TAT to nanoliposomes. TAT-CurcNL has a size range of 196.5 to 3.2 nm and was quantified using HPLC-MS/MS. Mass spectrometry, confocal microscopy, and a radioactivity assay using [3H]-sphingomyelin [42] all showed a threefold increase in nanoliposome absorption after TAT functionalization in human brain capillary endothelial cells (hCMEC and D3).

Mourtias also used the thin film hydration approach to make multifunctional nanoliposomes containing curcumin-lipid derivatives. When DSPE-PEG2000 was reacted with 4-methoxytrityl-thiol for the first time, it produced DSPEPEG2000-S-Mmt, which is the precursor to DSPE-PEG2000SH, a functionalized lipid available on the market. There were no by-products and the reaction proceeded quickly in the presence of DIPEA (Diisopropylethylamine) during the DSPE-PEG2000-S-Mmt synthesis [118]. A PEG spacer was added between curcumin and lipids to manufacture this derivative. When DSPEPEG2000-SH was deprotected with thiol, it reacted with curcumin to produce the DPSPEG2000-CURC derivative. Nanoliposome membranes were successfully included with this novel synthetic substance. For the second phenol-protons of the curcumin moiety, DIPEA was added and the resulting DIPEA salt was proved to effectively identify A deposits in post-mortem tissues of Alzheimer’s disease (AD) patients’ tissues. One of the best-selling herbal remedies, ginkgo *biloba,* has its origins in traditional Chinese medicine. Antioxidant activity in the CNS is observed to boost the activities of superoxide dismutase, catalase, glutathione peroxidase, and glutathione reductase. As a result of increased antioxidant activity in the hippocampi, Ginkgo *biloba* aids in memory and learning [119]. Non-ionic bilayer vesicles based on surfactants constitute niosomes. Unlike liposomes, niosomes may contain both hydrophilic and hydrophobic drugs in the same system [47,120]. It is possible that the niosomes containing medications for the treatment of CNS disorders may pass the BBB. In freeze-dried powder (661 nm) and spray-dried powder (680 nm), the size of niosomes is affected by the drying procedure. Spray-dried niosomes had a higher zeta potential than freeze-dried niosomes, on the other hand. Because the zeta potential of spray dried noisome powder is high, they are more stable, owing to the substantial electrostatic repulsion between particles [48,121].

#### 6.2.2. Green Synthesized Nanoparticles

Green chemistry—in fact, green technology—is currently being pushed for its use in nanotechnology as an eco-friendly option. However, for herbal extracts, metal nanoparticles have been shown to be more favourable than microorganisms in the creation of nanoparticles. The organic components may decompose into biodegradable material because of the presence of polyphenols. Shankhapushpi is n antihypertensive, immunomodulatory, and anticonvulsant ayurvedic plant. The antioxidant capability of the ayurvedic medicine Convolvulus pluricaulis was discovered to improve memory in nanoparticles made using iron oxide as a precursor. Researchers are still trying to understand the molecular process of these micro particles [122]. As an anti-epileptic, insect repellent, and antioxidant, the Pulicaria undulata plant is also often used in traditional Chinese medicine. The increased concentration of silver nanoparticles (AgNPs) in the brain leads to an increase in AgNPs’ interaction with proteins, which prevents fibril formation by decreasing protein conformation and self-association in Pulicaria undulata nanoparticles made with silver (AgNPs) [123].

## 7. Nanotechnology-Induced Bioinformatics for Early Diagnosis

In the last three decades, several computational programs have been continuously developed and applied to improve the quality of biomedicine in an interdisciplinary model. At the same time, the term bioinformatics, a combination of computational biology and mathematical algorithms, has been used for leading current biomedicine and biomedical research. Biological data-restoration (proteomics and genomics related to disease biology), biological data and text mining (analyses of massive amounts of data and text to restore biological data), and genomic-proteomic investigation (in the contest of mutation and drug resistance pathways and biomarkers) have been used to guide and solve several complex problems in biomedical areas through the wide range of cost and resource-saving technology. For example, the Human Genome Project and other OMICS (genomics, proteomics, metabolomics, and glycomics) projects have revolutionized bioinformatics support to biomedical research. Overall, integrated bioinformatics tools can gather and analyse massive amounts of biological and laboratory data to help understand diseases and select ideal therapies [124,125].

**Figure 2:**
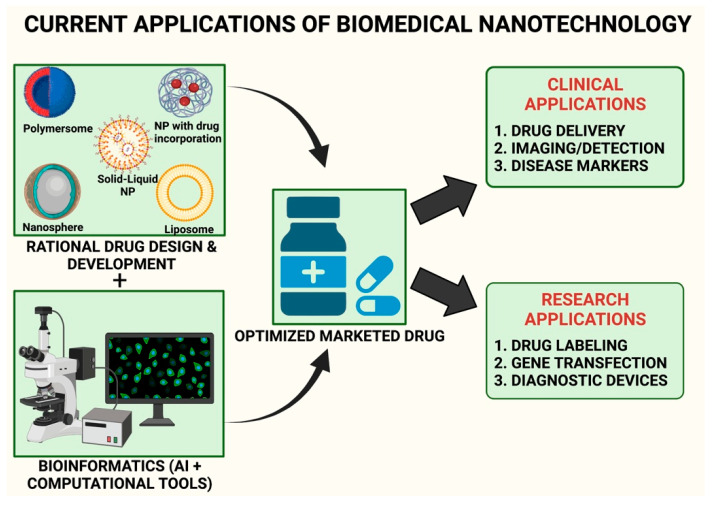
A schematic presentation of nano-informatic (nanotechnology and bioinformatics) in present dementia or neuro-disorder research.

Simultaneously, nanotechnology is another demanding sector in biomedicine for generating more prospective technological and technical discoveries in medication delivery. Strategically, when nanotechnology is merged with bioinformatics, the phrase used is ‘nano informatics’, and this discipline has more potential for attaining breakthrough analyses in biomedical drug development applications [126,127]. Figure 2 illustrates the role of nano informatics for the development of therapies for dementia. Currently, nano informatics have become an emerging field and have been accepted by health and regulatory bodies such as US NSF, NIH, the National Cancer Institute, and the European Commission, etc., for analysing and processing the structure and physiochemical characteristics of nanoparticles, their formulation, as well as for their application in the treatment of various diseases such as neurodegenerative disorders and cancer. The experimentation for NDs is more expensive and sensitive. Bioinformatics can be used for early detection, understanding the disease, and application, while nanotechnology can be used for studying the diagnosis or treatment strategy through the use of drug delivery that is target specific [125,127].

Dementia, a brain disorder which is characterized by memory loss, is a common disorder; AD is its most predominant form, accounting to 60–70% of dementia cases, and about 10 million cases have been recorded per year. Mostly, in brain disorders, the synaptic transmission is affected, and its target is the ionotropic glutamate receptor (iGluR), which belongs to the most poorly understood pharmacological target, the G-coupled receptor (GPCR). However, the current updated research involves analysing brain physiology and targeting amyloid plaques amyloid-beta neurofibrillary tangles, tau protein, astrocyte associated β-secretase, cholinergic neuron associated butyrylcholinesterase, cognitive function in medial temporal lobe cortex, secretase (presenilin I), central nerves system associated GPCR-target, and dopamine-2 receptor, etc., for Alzheimer and dementia treatment [128,129]. In drug development, there are more than a hundred pipeline drugs under clinical investigation [130,131].

In the history of drug discovery, most drugs have failed due to lack of consideration of pharmacokinetics. These drugs are unable to cross the blood-brain barrier (BBB) [132,133]. For a drug to be active and to show the desired effect, the drug should have the ability to penetrate the BBB. Most of these drugs, however, have been unable to cross and as a result show about <1% effectiveness as compared to the administered dose, or sometimes exert neurotoxicity. Therefore, the development of target-specific nano-drug delivery, which can pass through the BBB, can play an important role in the management and treatment of dementia or Alzheimer’s diseases [134,135]. Nevertheless, so far, the nano system is insufficient, and attempts are ongoing for the optimization of nano systems and drug applications in NDs [125,136]. Through the use of nano informatic tools, the interaction-cum-efficacy of proposed therapeutic agents with the target protein can be predicted along with obtaining relevant information on the BBB profile and possible neurotoxicity side effects that can be observed. [54,137,138,139,140]. For example, tools namely, admetSAR (http://lmmd.ecust.edu.cn/admetsar2/) (accessed on 10 November 2021), SwissADME (http://www.swissadme.ch/) (accessed on 10 November 2021), LightBBB (http://bioanalysis.cau.ac.kr:7030/) (accessed on 10 November 2021), as well as databases such as VariCarta (https://varicarta.msl.ubc.ca/index) (accessed on 10 November 2021), AlzGene (http://www.alzgene.org/) (accessed on 10 November 2021), and NDDVD (http://bioinf.suda.edu.cn/NDDvarbase/LOVDv.3.0) (accessed on 10 November 2021) have been used to obtain large data sets of neurological therapeutics. Overall, nano informatics could be a commanding approach towards exploring and understanding mechanisms along with locating and developing better suited and more efficacious therapeutic agents in a cost-effective manner. Table 3 summarizes the miRNAs used for treatment of neurological disorders.

## 8. Limitations of Nanotechnology-Based Approaches for ND

Using nanotheranostic techniques to treat neurodegenerative illnesses, as discovered by Indrasekara et al. [165], have their limits because each patient has a unique genome and neurological functioning and features, and so no one strategy can be employed to treat all of them. Every person must be diagnosed and treated for these neurodegenerative conditions individually, which is a difficult undertaking and does not aid in establishing one approach or treatment methodology that works for everyone [166]. (De Lau et al., 2006).

In addition, Kim et al. found that if the injection is not performed properly, the NPs utilised in this approach may be absorbed by the blood or other body parts instead of the intended target location [167]. This is a difficulty or restriction that has been highlighted by Kim et al. If the medicine is not injected properly, it will not be absorbed or utilised to treat the intended location for treating neurodegenerative illnesses such as Alzheimer’s disease and Parkinson’s disease. Because just a little amount of the medication actually makes its way to where it is needed, some have contended that it has not yet been shown that the therapy is 100% successful [168].

In order to progress this study into viable therapeutic medicines, it is critical that safety concerns be addressed. As a reminder, the most successful NP formulations for the brain delivery nonetheless accumulate extensively in other parts of the body, such as the spleen, the liver, and the kidney. As a result, it is critical to create nanoformulations that are only activated when they reach the brain [169] instead of activating when they reach other parts of the body. Nanoparticles in regenerative medicine may soon benefit from advances in triggerable nanoformulation technology. The production of nanoparticles that target particular brain cells is a significant problem that warrants more study. Targeting particular brain cells such as dopaminergic neurons (the primary target in Parkinson’s disease), microglia (neuroinflammation), or neural stem cells (neuronal repair) may boost its potential therapeutic usefulness in the context of neurodegenerative illnesses.

Multifunctionality is required for brain-targeting NP because of the BBB’s protective role, which makes it challenging to target molecules to the brain parenchyma. As a matter of fact, the BBB’s primary job is to protect brain tissue from potentially hazardous chemicals. NPs neurotoxicity must be examined for the same reasons as a more traditional medicinal system’s neurotoxicity. Microglial activation is a critical consideration in NP neurotoxicity because it plays a role in the neurodegenerative pathogenic process in the vast majority of CNS illnesses. When using cultured microglial cells, an in vitro study found that TiO_2_ NPs and HAP (hydroxyapatite) NPs were able to trigger inflammation-related iNOS (inducible nitric oxide synthase) and activate the NF-B signalling pathway. All the tested NPs induced, to a variable extent, the increase in pro-inflammatory molecules [170]. It was evidenced that TiO_2_ and HAP NPs may activate microglial cells and lead to probable pathogenic alterations in the olfactory bulb, striatum, and hippocampal regions of mice, concluding that these inorganic NPs contributed to the dysfunction and cytotoxicity in PC12 cells.

## 9. Future Nano Therapeutics for ND

Without a doubt, precision-based nanomedicine will gain more attention in the coming years. However, despite advances, the use of phages in humans has yet to gain widespread acceptance. There is a gap that must be bridged between animal trials and human use. Approval from the Food and Drug Administration is required, and it is likely to follow on the heels of increasingly promising research on phage-based nanomedicine [171].

Exosomes, which are naturally produced by human cells, are emerging as a new generation of highly protective nanoplatforms for efficient drug delivery [172]. They can transport not only therapeutics but also molecular imaging agents for use in precision therapeutics and diagnostics. Exosomes are an ideal nanoplatform for loading both hydrophilic and lipophilic agents due to their unique structure, which includes an aqueous core and a membrane rich in lipid rafts.

Nonetheless, despite the current promising research on exosomes, several major challenges remain to be overcome, including a lack of characterization of exosomes derived from different sources, low exosomal yield, and encapsulation efficiency, and a lack of advanced purification techniques with high efficiency. Exosome molecular and nanoengineering will provide insights into future effective and precision medicine for devastating diseases such as neurodegenerative disorders. Because targeted exosomal delivery is a rapidly developing field, aptamer-mediated exosomal delivery is becoming appealing for the development of smart nano-delivery systems due to its ease of use, high performance at the nanoscale, enhanced efficacy, safety, and low cost [173,174].

Although preclinical studies have provided the biological and mechanistic foundation for previous clinical trials, future efforts to improve the predictive accuracy of preclinical studies will be critical to clinical trial success. To improve clinical translation, health experts hope to develop research guidelines such as the Stroke Therapy Academic Industry Roundtable (STAIR) recommendations. The Federal Interagency Traumatic Brain Injury Research (FITBIR) system was designed to share TBI-related research across the research community [175]. Future efforts for the development of nanomaterials to treat acute brain injuries include accurate measurement of nanomaterial pharmacokinetics in living organisms, careful selection of animal models that recapitulate specific human pathology relevant to nanomaterial design and payload, designing experiments that consider multiple biological variables, and the development of technology to quantitatively measure biomarkers that can accurately predict outcomes in humans.

## 10. Conclusions

Nanotechnology can alter neuroscience-based information and restorative methodologies, and can be used to possibly make significant commitments for the development of nano-empowered medication for the treatment of NDs. Equal advancements in neurophysiology and neuropathology exploration would help in the advancement in nanotechnology, which can be used to provide CNS recovery and neuroprotection. Accordingly, for utilization of nanotechnology in neural system science and neurosurgery, key factors that require consideration include: (1) breakthrough discoveries and developments in drug science and material science, which can help in the manufacturing of the described methodologies; (2) development and advancement of sub-atomic science, sensory system-based neurophysiology, and neuropathology; and (3) planning and combination of explicit nano-empowered therapies to the CNS, which exploit the initial two factors. As a result, nanotechnology could provide the solution and can offer breakthrough therapies for the management and treatment of NDs and can also be used to bypass the current problem of available neurological therapies i.e., non-specific targeting and lower efficacy rates of drug therapies. Therefore, taken together, neurosurgeons, nervous system specialists, neuroscientists, and drug researchers and architects, should take part in utilizing the power of nanotechnology for drug delivery. Consistent with the profoundly interdisciplinary nature of this space of exploration, it is additionally significant to note that nano-informatics and nanotechnology can also provide innovative headways and progressions that are related to fundamental and clinical neuroscience.

## Figures and Tables

**Table 1:** Summary of the various conventional strategies used for the treatment of AD and PD.

Strategy	Alzheimer’s Disease	Parkinson Disease
**Modulation of neurotransmitters (approved therapies)**	Acetylcholinesterase inhibitors NMDA antagonists	Precursors of Dopamine MAO-B inhibitors COMT inhibitors Dopaminergic agonists Anti-cholinergics
**Disease modifying therapies (under investigation)**	1. **Amyloid based therapy** Secretase modulation Amyloid aggregate prevention Amyloid clearance promotor 2. **Tau-based therapy** Tau hyperphosphorylation inhibition Tau protein degradation Tau oligomerization inhibition	1. **α-Synuclein-based therapy** Amyloid aggregate prevention α-synuclein fibril formation blockade Modulation of α-synuclein related lipidome 2. **Non-dopaminergic therapy** Antagonists of adenosine receptor Antagonists of NMDA Agonists of Glucagon such as peptide-1
**Immunotherapy**	Passive immunization Solanezumab Crenezumab Active immunization CAD106	Passive immunization Solanezumab Active immunization PD01A
**Gene-based therapy**	Regulation of presenilin expression	Expression of synapsin 3 modulation
**Other**	ROS reduction, oxidative stress reduction Anti-inflammatory agents Caspase inhibitors Metal chelators, Statins PPAR-γ (Peroxisome proliferator-activated receptor-γ)	Anti-inflammatory agents Melatonin Nicotine Calcium channel blockers Antioxidants Iron chelators

**Table 2:** Summary of the materials used, active moiety entrapped, and outcomes obtained upon administration of NPs.

Material	Name of Active Moiety Entrapped	Size	Outcome of Study	Reference
Cholesterol	α-bisabolol	139.5 nm	NPs drastically decrease free radical generation, lower β-secretase, caspase-3, cholinesterase, and Bax expression, and increase Bcl-2 protein expression.	[104]
Cetyl palmitate miglyol-812	Quercetin	200 nm	It is non-toxic to hCMEC/D3 cells and penetrates the BBB more so than free drug. NPs also prevent A peptide fibril production.	[105]
Caprylic and capric triglycerides, sorbitan monostearate	Curcumin	247 nm	Curcumin-NPs protect against A42-induced behavioural and neurochemical alterations in AD mice model.	[106]
Cholesterol	α-bisabolol	Not reported	Inhibited A aggregation and protected Neuro-2a cells from A-induced neurotoxicity.	[104]
PLGA	Curcumin	150–200 nm	Curcumin encapsulated-PLGA nanoparticles, destroyed amyloid aggregates, exhibited an anti-oxidative property, and are non-cytotoxic.	[107]
DSPE-PEG2000-MAL	Quercetin	200 nm	After 4 h, RVG29-nanoparticles had 1.5 times the permeability across the blood-brain barrier compared to non-functionalized nanoparticles.	[108]
PEG, PLGA	Epigallocatechin-3-gallate	100 nm	GCG and AA NPs resulted in a marked increase in synapses, as judged by synaptophysin (SYP) expression, and reduction of neuroinflammation as well as amyloid β (Aβ) plaque burden and cortical levels of soluble and insoluble Aβ(1–42) peptide.	[109]
poly(ethylene glycol)-co-poly(ε-caprolactone)	Ginkgolide B	91 nm	NPs facilitated the sustained release of GB into the blood, thereby improving its ability to accumulate in the brain and to treat PD.	[110]
chitosan poly ethyleneglycol-poly lactic acid	Acteoside	100 nm	Significantly reversed dopaminergic (DA) neuron loss in the substantia nigra and striatum of sick mice.	[111]
Monomethoxy polyethylene glycol	Apomorphine (AMP)	100 nm	The encapsulation of AMP into the nanoparticles inhibits oxidization. The intranasal administration of the AMP-loaded nanoparticles transports AMP across the BBB.	[112]

**Table 3:** List of miRNAs used as therapeutics (conventional and nonconventional) for some NDDs. The investigated drug, the miRNAs, their sources, the disease state as well as the references are indicated.

Drug	miRNA	Source of miRNA	Disease State Used	Reference
**Donepezil**	miRNA-206-3p	Mouse-Hippocampus, cortex	Dementia	[141]
**Simvastatin**	miRNA-106b	SH-SY5Y cells; Mice brain tissue-APP/PS1		[142]
**Osthole**	miRNA-9	overexpressed APP cells	Alzheimer’s Disease (AD)	[143,144]
	miRNA-107	Overexpressed APP cells Mice brain tissue-APP/PS1		[143]
	miRNA-101a-3p			[143]
**AGR** **-** **GRg1**	miRNA-873-5p	Mouse Hippocampus		[145]
**L-Dopa**	miRNA-30b-5p, miRNA-30a-5p	Plasma	PD	[146]
	miRNA-29a-3p, miRNA-30b-5p, miRNA-103a-3p	Peripheral Blood mononuclear cells (PBMC’s)		[147]
	miRNA-16-2-3p, miRNA-26a-2-3p, miRNA-30a	Peripheral blood		[148]
	miRNA-155	PBMCs		[149]
**L-Dopa, Amantadine**	miRNA-7, miRNA-9-3p, miRNA-9-5p	Peripheral blood		[150]
**Interferon-β**	miRNA-29	PBMCs	Multiple Sclerosis	[151]
	miRNA-145	Whole blood		[152]
	miRNA-29b-3p	PBMCs		[153]
	miRNA-326			[154]
	miRNA-26a-5p			[155]
	miRNA-146a			[156]
**Natalizumab**	miRNA-150 CSF,	Plasma		[157]
	miRNA-126, miRNA-17	CD4 + T cells		[158,159]
	miRNA-17~92, miRNA-106b~25	B lymphocytes		[160]
	miRNA-26a, miRNA-155	PBMCs		[153]
	miRNA-155	Monocytes		[161]
**Dimethyl fumarate**	miRNA-155	Monocytes		[162]
**Fingolimod**	miRNA-150	Plasma		[163]
	miRNA-23a	Whole Blood		[164]
**Natalizumab**	miRNA-320, miRNA-320b, miRNA-629	Blood	Progressive multifocal leukoencephalopathy	[163]

## Abbreviations

BBB	Blood brain barrier
CNS	Central nervous system
ND	Neurological and neurodegenerative diseases
NP	Nanoparticle
AD	Alzheimer’s disease
PD	Parkinson’s disease
ALS	Amyotrophic horizontal sclerosis
JNK	c-Jun N-terminal kinase
AChE	Acetylcholinesterase
IC50	Half-maximal inhibitory concentration
SAC	S-allyl cysteine
MPP	Phenyl pyridinium
SOD	Superoxide dismutase
6-OHDA	6-hydroxydopamine
CAT	Chloramphenicol acetyltransferase
GPx	Gluthathione peroxidase
GPR	Glutathione reductase
ATP	Adenosine triphosphate
iNOS	Inducible nitric oxide synthase
COX-2:	Cyclo-oxygenase 2
MDA	Malondialdehyde
GSH	Glutathione
CPPME	Celastrus paniculate wild whole plant methanolic extract
LPO	Lipid peroxidation
DPPH	2,2-diphenyl-1-picrylhydrazyl
GABA_A_	Gamma- amino butyric acid
MAO	Monoamine oxidases
DNA	Deoxyribo nucleic acid
mRNA	Messenger ribonucleic acid
5-HT	5-hydroxytrptamine/serotonin
DA	Dopamine
ROS	Reactive oxygen species
VMAT	Vesicular monoamine transporter
NMDA	N-methyl-D-aspartic acid
COMT	Catechol ortho-methyl transferase
CSF	Cerebrospinal fluid
PET	Positron emission tomography
SPECT	Single photon emission computed tomography
NIH	National Institute of Health
NSF	National Science Foundation
USFDA	United States Food and Drug Administration
STAIR	Stroke Therapy Academic Industry Roundtable
FITBIR	Federal Interagency Traumatic Brain Injury Research
